# Sentinel node navigation surgery for gastroduodenal neuroendocrine tumors: Two case reports

**DOI:** 10.1097/MD.0000000000004063

**Published:** 2016-07-01

**Authors:** Takaaki Arigami, Yoshikazu Uenosono, Shigehiro Yanagita, Keishi Okubo, Takashi Kijima, Daisuke Matsushita, Masahiko Amatatsu, Takahiko Hagihara, Naoto Haraguchi, Yuko Mataki, Katsuhiko Ehi, Sumiya Ishigami, Shoji Natsugoe

**Affiliations:** a Department of Digestive Surgery, Breast and Thyroid Surgery, Field of Oncology; b Molecular Frontier Surgery, Course of Advanced Therapeutics, Kagoshima University Graduate School of Medical and Dental Sciences, Kagoshima, Japan.

**Keywords:** gastroduodenal neuroendocrine tumors, lymph node metastasis, near-infrared fluorescence imaging, sentinel node navigation surgery

## Abstract

The percentage of gastroduodenal neuroendocrine tumors (NETs) among all gastroenteropancreatic (GEP) NETs has gradually increased worldwide. Sentinel node navigation surgery (SNNS) has been developed as a personalized approach in the surgical strategy for early gastrointestinal tract cancers. We herein report 2 cases of gastroduodenal NETs treated with SNNS.

^99m^ Technetium-tin colloid including indocyanine green was endoscopically injected into the submucosa around a tumor the day before surgery. Basin dissection including the sentinel nodes (SNs), which were identified by Navigator GPS and near-infrared fluorescence imaging, was performed during laparoscopic surgery. SNs were intraoperatively examined using hematoxylin–eosin (HE) staining.

SNs were detected in 2 patients. Lymph node metastasis was intraoperatively identified in 1 of the 2 patients. Consequently, 1 patient with metastatic SNs underwent laparoscopic gastrectomy with lymphadenectomy. Pathological findings identified submucosal NET measuring 6.0 mm × 5.0 mm.

Our results suggest that SNNS is a promising surgical tool for detecting subclinical lymph node metastasis in patients with gastroduodenal NETs.

## 1 Introduction

In the 2010 WHO classification for neuroendocrine neoplasms (NENs), gastroenteropancreatic (GEP) NENs have been classified into the following 3 categories: Grade 1 (G1): low-grade neuroendocrine tumors (NETs), Grade 2 (G2): intermediate-grade NETs, and Grade 3 (G3): high-grade neuroendocrine carcinomas.^[1]^ This grading system is strictly determined by mitotic count values and the Ki-67 index.^[1]^ Gastroduodenal NETs are a type of GEP–NET, and the incidence of gastric NETs among all GEP–NETs is known to range between 5.0% and 14.6%.^[2]^ The incidence of gastroduodenal NETs has gradually increased worldwide.^[2]^ Rindi et al^[3]^ examined and classified 55 patients with gastric NETs into 3 clinicopathological subtypes. Gastric NETs with types 1 and 2 are associated with hypergastrinemia, while gastric NETs with type 3 exhibit sporadic properties under normal gastrin levels.^[3,4]^ According to the criteria of the National Comprehensive Cancer Network (NCCN) Clinical Practice Guidelines in Oncology (version 1.2015), observation or endoscopic resection is recommended as an initial treatment in cases of type 1 locally gastric NETs with a tumor size ≤2.0 cm.^[5]^ These guidelines also suggest endoscopic or surgical resection for cases of type 1 locally gastric NETs with a tumor size >2.0 cm.^[5]^ On the other hand, cases of type 3 locally gastric NETs are at risk of lymph node metastasis due to the aggressive behavior of this tumor subtype.^[4]^ Consequently, the NCCN guidelines recommend radical gastrectomy with lymph node dissection for cases of type 3 locally gastric NETs.^[5]^ These guidelines also suggest endoscopic or wedge resection as an optional treatment for cases of type 3 locally gastric NETs measuring ≤2.0 cm in diameter.^[5]^ However, previous studies have detected lymph node metastasis in cases of type 1 gastric NETs measuring ≤2.0 cm or duodenal NETs measuring ≤5.0 mm in diameter.^[6,7]^ These findings indicate an equivocal therapeutic strategy for gastroduodenal NETs.

Sentinel node navigation surgery (SNNS) has recently been developed as a minimally invasive tool with personalized lymphadenectomy for early gastrointestinal tract cancers, including gastric cancer.^[8–10]^ A prospective multicenter trial in Japan demonstrated the clinical safety and utility of SNNS using the endoscopic dual tracer method for early gastric cancer measuring <4.0 cm in diameter.^[10]^ According to this prospective study consisting of 397 patients with cT1–T2 tumors preoperatively, the SN detection and accuracy rates were 97.5% and 99.0%, respectively.^[10]^ Moreover, we reported the potential of SNNS as a tool for grasping the lymph node metastatic status in patients with early gastric cancer after noncurative endoscopic resection.^[11]^ However, the clinical utility of SNNS for gastroduodenal NETs has not yet been established. We herein report 2 cases of gastroduodenal NETs treated with SNNS.

## 2 Case report

In this case report, anatomical definitions of dissected lymph node stations were classified based on the criteria of the Japanese classification of gastric carcinoma (3rd English edition).^[12]^ The Ethics Committee at Kagoshima University approved this study and all patients provided written informed consent to participate in all procedures associated with the study.

### 2.1 Case 1

**Figure 1:**
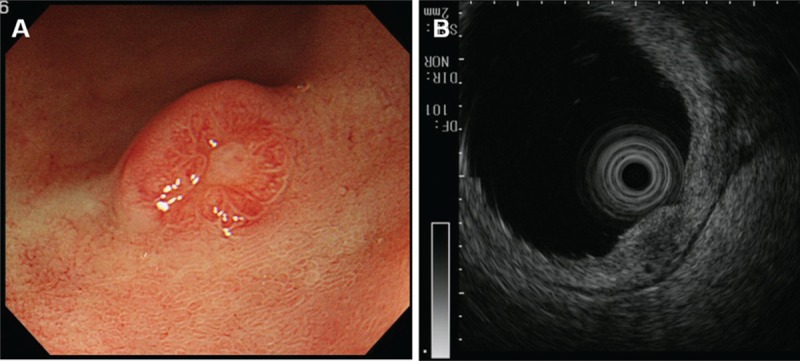
Preoperative examinations by (A) esophagogastroduodenoscopy and (B) endoscopic ultrasonography. Case 1 had a 5.0-mm submucosal NET on the duodenal bulb.

A 77-year-old woman was admitted to our hospital for the treatment of a 5.0-mm submucosal NET diagnosed by endoscopic biopsy specimens on the duodenal bulb (Fig. 1 A and B). The patient was clinically diagnosed as being free of lymph node metastasis by ultrasonography (US) and computed tomography (CT) prior to surgery.

**Figure 2:**
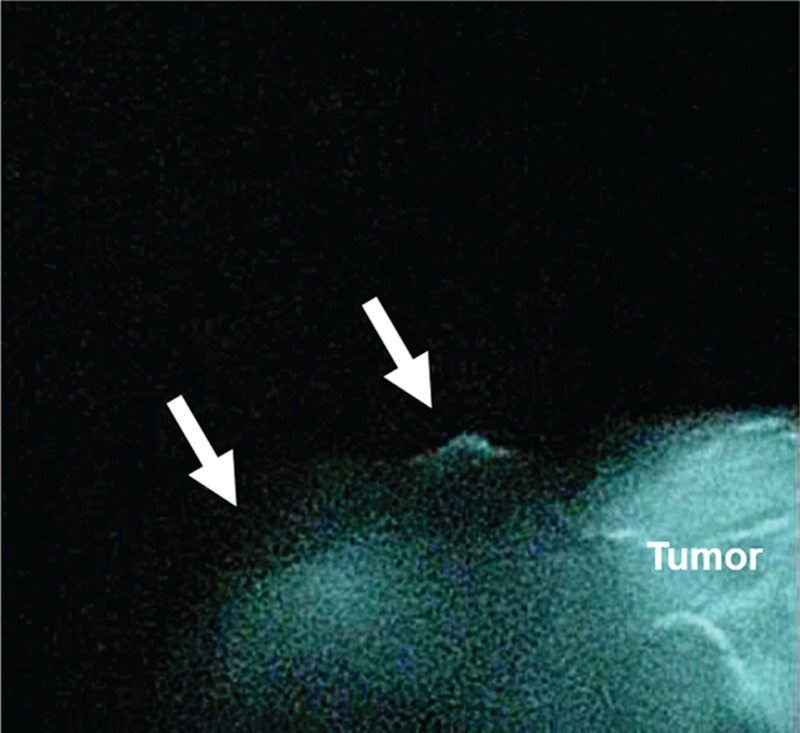
Laparoscopic findings based on near-infrared fluorescence imaging. Cases 1 had 2 sentinel nodes in station No. 6 (arrow).

**Figure 3:**
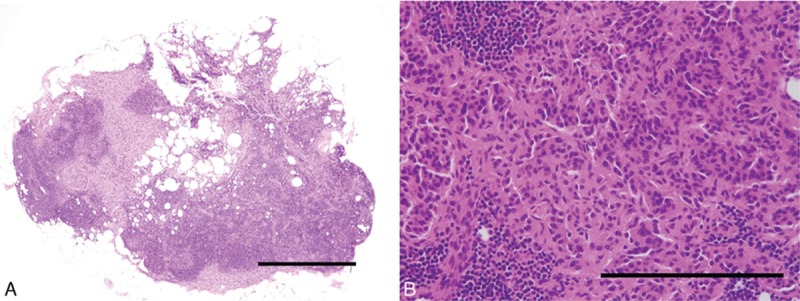
Hematoxylin–eosin staining of a sentinel node. (A) The scale bar indicates 500 μm. Original magnification ×40. (B) The scale bar indicates 200 μm. Original magnification ×400.

One day before surgery, 4 mCi (2 mL) of ^99m^ technetium-tin colloid including indocyanine green was endoscopically injected into the submucosa around the tumor at 4 sites using a disposable 23-gauge needle (MAJ-75, Olympus Corp., Tokyo, Japan). Two sentinel nodes (SNs) were identified in station No. 6 by the laparoscopic system with near-infrared fluorescence imaging (Olympus Corp.) (Fig. 2). Therefore, basin dissection including these SNs was performed. Radioisotope (RI) uptake was counted by Navigator GPS (TYCO HEALTHCARE, Ltd, Tokyo, Japan) in all basin-dissected lymph nodes. The 2 SNs that absorbed a 10-fold greater amount of RI than the background level were confirmed in station No. 6. Intraoperative hematoxylin–eosin (HE) staining revealed one metastatic SN in station No. 6 (Fig. 3 A and B). Therefore, the operative method was converted to laparoscopic-assisted distal gastrectomy with D2 lymphadenectomy based on the Japanese Gastric Cancer Treatment Guidelines 2010 (ver. 3).^[13]^

**Figure 4:**
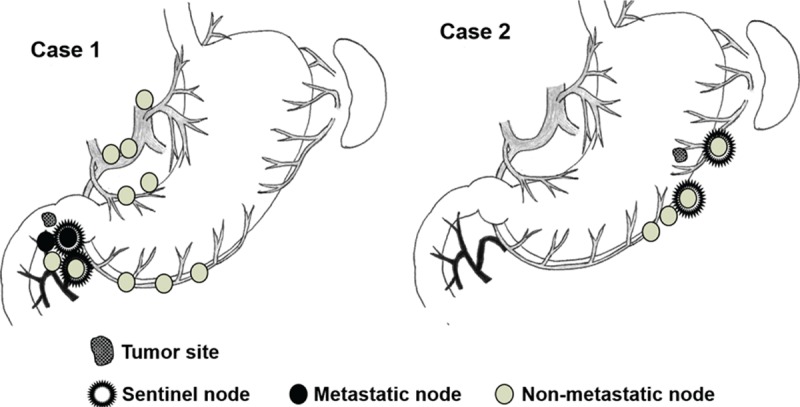
Sentinel node mapping in patients with gastroduodenal neuroendocrine tumors. Sentinel nodes contained tumor cells in Case 1.

Pathological HE findings revealed submucosal NET measuring 6.0 mm × 5.0 mm with lymphatic invasion in the duodenum and two metastatic lymph nodes in station No. 6 as SNs and non-SNs, respectively (Fig. 4). Furthermore, this patient had low-grade NET (G1) with one mitosis per 10 high-powered fields and a Ki-67 index <2.0%.

### 2.2 Case 2

A 65-year-old woman was admitted for the treatment of a 7.0-mm submucosal type 1 NET diagnosed by endoscopic biopsy specimens on the middle third region of stomach. The patient was preoperatively diagnosed as being free of lymph node metastasis by US and CT.

SNNS was performed as described for Case 1. The patient had 2 SNs in station No. 4sb and No. 4d (Fig. 4). An intraoperative assessment based on HE staining did not show lymph node metastasis in these SNs. Therefore, this patient underwent laparoscopic local resection without lymphadenectomy as the clinical application of SNNS.

Pathological HE examinations revealed submucosal G1 NET measuring 4.0 mm × 4.0 mm without lymphatic invasion. Metastatic lymph nodes were not identified in any basin-dissected lymph node including SNs and non-SNs.

## 3 Discussion

We herein reviewed 2 cases of gastroduodenal NETs for which SNNS was performed. To the best of our knowledge, we are the first to describe the clinical utility of SNNS as a personalized surgical treatment for gastroduodenal NETs.

According to the NCCN guidelines, endoscopic or wedge resection without lymphadenectomy is basically indicated for local gastroduodenal NETs measuring ≤2.0 cm.^[5]^ Endoscopic treatments are strongly recommended for duodenal NETs.^[5]^ Transduodenal local excision with or without lymph node sampling and pancreatoduodenectomy are ranked as optional surgeries in the initial treatment of nonmetastatic duodenal NETs.^[5]^ This proposal may depend on the invasiveness of surgery for patients with duodenal NETs. However, lymphadenectomy is understandably needed for patients with lymph node metastasis. It is clinically difficult to diagnose lymph node metastasis using preoperative imaging examinations, such as US, CT, and positron emission tomography, in patients with gastroduodenal NETs. In the present study, Case 1 had metastatic SN measuring <3.0 mm at the greatest dimension and it was not possible to identify this metastatic SN by preoperative imaging examinations. Consequently, it is important to assess the presence or absence of lymph node metastasis intraoperatively using pathological examinations. An intraoperative diagnosis of lymph node metastasis may contribute greatly to the development of further surgical treatments with personalized lymphadenectomy for patients with gastroduodenal NETs.

A clinical application based on SNNS has recently been initiated for patients with gastrointestinal tract cancers, and several investigators have reported the clinical utility of SNNS for early gastric cancer.^[8–10]^ The application of SNNS to patients with gastroduodenal NETs may represent the most promising surgical procedure for these patients. Although Cases 1 had 2 metastatic lymph nodes, SNs included 1 metastatic lymph node. Cases 1 and 2 described herein are currently alive without disease recurrence 77 and 16 months after surgery, respectively. These results suggest that the SN concept may be established for gastroduodenal NETs as well as gastric cancer. Accordingly, endoscopic or wedge resection may be recommended for patients with pathologically nonmetastatic SNs, while curative resection with lymph node dissection may be performed in order to prevent lymph node recurrence in patients with pathologically metastatic SNs.

The laparoscopic system used herein in combination with near-infrared fluorescence imaging was introduced to identify SNs in addition to a radioguided method. Recent advances in near-infrared fluorescence imaging systems have simplified the visualization of SNs and effluent lymphatic vessels from primary tumor sites in SNNS.^[14–17]^ Although it was not possible to detect SNs under a normal view in Case 1, we easily identified 2 SNs in station No. 6 using the near-infrared fluorescence imaging system with indocyanine green. Furthermore, the combination of this imaging system and a radioguided approach is recommended as a dual-tracer method to decrease the false-negative rate in SNNS.

In conclusion, we herein demonstrated that SNNS has potential in personalized lymphadenectomy for patients with gastroduodenal NETs. This is a preliminary report to review the clinical utility of SNNS for gastroduodenal NETs. Therefore, further large prospective studies are needed in order to strengthen our conclusion.

## References

[1] Bosman FT Carneiro F Hruban RH World Health Organization (WHO) Classification of Tumours of the Digestive System. 4th ed Geneva, Switzerland: WHO Press; 2010.

[2] Fraenkel M Kim MK Faggiano A Epidemiology of gastroenteropancreatic neuroendocrine tumours. *Best Pract Res Clin Gastroenterol* 2012; 26:691–703. 23582913 10.1016/j.bpg.2013.01.006

[3] Rindi G Luinetti O Cornaggia M Three subtypes of gastric argyrophil carcinoid and the gastric neuroendocrine carcinoma: a clinicopathologic study. *Gastroenterology* 1993; 104:994–1006. 7681798 10.1016/0016-5085(93)90266-f

[4] Rindi G Bordi C Rappel S Gastric carcinoids and neuroendocrine carcinomas: pathogenesis, pathology, and behavior. *World J Surg* 1996; 20:168–172. 8661813 10.1007/s002689900026

[5] Kulke MH Shah MH Benson AB III Neuroendocrine tumors, version 1.2015. *J Natl Compr Canc Netw* 2015; 13:78–108. 25583772 10.6004/jnccn.2015.0011

[6] Soga J Endocrinocarcinomas (carcinoids and their variants) of the duodenum. An evaluation of 927 cases. *J Exp Clin Cancer Res* 2003; 22:349–363. 14582691

[7] Grozinsky-Glasberg S Thomas D Strosberg JR Metastatic type 1 gastric carcinoid: a real threat or just a myth? *World J Gastroenterol* 2013; 19:8687–8695. 24379587 10.3748/wjg.v19.i46.8687 PMC3870515

[8] Arigami T Natsugoe S Uenosono Y Evaluation of sentinel node concept in gastric cancer based on lymph node micrometastasis determined by reverse transcription-polymerase chain reaction. *Ann Surg* 2006; 243:341–347. 16495698 10.1097/01.sla.0000201453.65534.f1 PMC1448932

[9] van der Zaag ES Bouma WH Tanis PJ Systematic review of sentinel lymph node mapping procedure in colorectal cancer. *Ann Surg Oncol* 2012; 19:3449–3459. 22644513 10.1245/s10434-012-2417-0

[10] Kitagawa Y Takeuchi H Takagi Y Sentinel node mapping for gastric cancer: a prospective multicenter trial in Japan. *J Clin Oncol* 2013; 31:3704–3710. 24019550 10.1200/JCO.2013.50.3789

[11] Arigami T Uenosono Y Yanagita S Feasibility of sentinel node navigation surgery after noncurative endoscopic resection for early gastric cancer. *J Gastroenterol Hepatol* 2013; 28:1343–1347. 23663136 10.1111/jgh.12269

[12] Japanese Gastric Cancer Association. Japanese classification of gastric carcinoma: 3rd English edition. *Gastric Cancer* 2011; 14:101–112. 21573743 10.1007/s10120-011-0041-5

[13] Japanese Gastric Cancer Association. Japanese gastric cancer treatment guidelines 2010 (ver. 3). *Gastric Cancer* 2011; 14:113–123. 21573742 10.1007/s10120-011-0042-4

[14] Cahill RA Anderson M Wang LM Near-infrared (NIR) laparoscopy for intraoperative lymphatic road-mapping and sentinel node identification during definitive surgical resection of early-stage colorectal neoplasia. *Surg Endosc* 2012; 26:197–204. 21853392 10.1007/s00464-011-1854-3

[15] Gilmore DM Khullar OV Gioux S Effective low-dose escalation of indocyanine green for near-infrared fluorescent sentinel lymph node mapping in melanoma. *Ann Surg Oncol* 2013; 20:2357–2363. 23440551 10.1245/s10434-013-2905-x

[16] Schaafsma BE Verbeek FP Rietbergen DD Clinical trial of combined radio- and fluorescence-guided sentinel lymph node biopsy in breast cancer. *Br J Surg* 2013; 100:1037–1044. 23696463 10.1002/bjs.9159 PMC3681835

[17] Schols RM Connell NJ Stassen LP Near-infrared fluorescence imaging for real-time intraoperative anatomical guidance in minimally invasive surgery: a systematic review of the literature. *World J Surg* 2015; 39:1069–1079. 25522896 10.1007/s00268-014-2911-6

